# Supplementary material for: Ferromagnetic Double Perovskite Semiconductors with Tunable Properties
Source: Adv Sci (Weinh). 2022 Jan 20;9(8):2104319. doi: 10.1002/advs.202104319 (PMC8922134; doi:10.1002/advs.202104319)
Supplement: Supplementary file 1 — Supporting Information [file ADVS-9-2104319-s001.pdf]

ADVANCED SCIENCE
Open Access

Supporting Information

for *Adv. Sci.*, DOI: 10.1002/advs.202104319

Ferromagnetic Double Perovskite Semiconductors with Tunable Properties

Lun Jin, Danrui Ni, Xin Gui, Daniel B. Straus, Qiang Zhang and Robert J. Cava**

Supporting Information

Ferromagnetic Double Perovskite Semiconductors with Tunable Properties

Lun Jin, Danrui Ni, Xin Gui, Daniel B. Straus, Qiang Zhang and Robert J. Cava**

L. Jin, D. Ni, X. Gui, D. B. Straus and R. J. Cava

Department of Chemistry, Princeton University, Princeton, NJ, 08544, USA

***** E-mails of corresponding authors: ljin@princeton.edu; rcava@princeton.edu

Q. Zhang

Neutron Scattering Division, Oak Ridge National Laboratory, Oak Ridge, TN, 37831, USA

Table of Contents

1. Rietveld refinement of ambient temperature neutron powder diffraction data

Figure S1. Observed (blue), calculated (green) and difference (cyan) plots from the Rietveld refinement of Sr_2GaSbO_6 (space group *I*4/*m*) against neutron powder diffraction data at 300 K.

Table S1. Structural parameters and crystallographic positions from the refinement of neutron powder diffraction data collected from Sr_2GaSbO_6 at 300 K.

Table S2 Selected bond lengths for the double perovskites from the Rietveld refinement of the Neutron diffraction data.

2. Scanning Electron Microscope Characterization

Figure S2. The FEI XL30 field-emission gun scanning electron microscope (SEM) photos of particle morphology of (a) $Sr_2Ga_{0.9}Mn_{0.1}SbO_6$, (b) $Sr_2Ga_{0.6}Mn_{0.4}SbO_6$ and (c) $Sr_2Ga_{0.1}Mn_{0.9}SbO_6$.

Figure S3. The scanning electron microscope (SEM) mapping results for (a) $Sr_2Ga_{0.9}Mn_{0.1}SbO_6$, (b) $Sr_2Ga_{0.6}Mn_{0.4}SbO_6$ and (c) $Sr_2Ga_{0.1}Mn_{0.9}SbO_6$.

3. Magnetization data collected from the $Sr_2Ga_{1-x}M_xSbO_6$ series (M = Cr/Mn/Fe)

Table S3. The Curie constant and Weiss temperature extracted from the fitting of paramagnetic susceptibility to the Curie-Weiss law, the observed effective moment per formula unit, and the calculated effective moment per formula unit predicted based on the spin-only formula for each composition of the Mn-doped double perovskite Sr_2Ga_{1-}

${}_{x}Mn_{x}SbO_6$ series and single perovskite $LaGa_{1-x}Mn_{x}O_3$ series (scaled based on its equivalent double perovskite formula).

Figure S4. The magnetization data collected from $Sr_2Ga_{0.9}Mn_{0.1}SbO_6$.

Figure S5. The magnetization data collected from $Sr_2Ga_{0.8}Mn_{0.2}SbO_6$.

Figure S6. The magnetization data collected from $Sr_2Ga_{0.7}Mn_{0.3}SbO_6$.

Figure S7. The magnetization data collected from $Sr_2Ga_{0.6}Mn_{0.4}SbO_6$.

Figure S8. The magnetization data collected from $Sr_2Ga_{0.5}Mn_{0.5}SbO_6$.

Figure S9. The magnetization data collected from $Sr_2Ga_{0.4}Mn_{0.6}SbO_6$.

Figure S10. The magnetization data collected from $Sr_2Ga_{0.3}Mn_{0.7}SbO_6$.

Figure S11. The magnetization data collected from $Sr_2Ga_{0.2}Mn_{0.8}SbO_6$.

Figure S12. The magnetization data collected from $Sr_2Ga_{0.1}Mn_{0.9}SbO_6$.

4. **Magnetization data collected from the $La_2Ga_{2-2x}Mn_{2x}O_6$ series**

Figure S13. The magnetization data collected from $LaGa_{1.8}Mn_{0.2}O_6$.

Figure S14. The magnetization data collected from $LaGa_{1.6}Mn_{0.4}O_6$.

Figure S15. The magnetization data collected from $LaGa_{1.4}Mn_{0.6}O_6$.

Figure S16. The magnetization data collected from $LaGa_{1.2}Mn_{0.8}O_6$.

Figure S17. The magnetization data collected from $LaGa_{1.0}Mn_{1.0}O_6$.

5. **The calculated band gaps for all the double perovskites prepared in this study**

Table S4. The calculated band gaps from Tauc plot by using both indirect and direct transition equation for all the double perovskites prepared in this study.

6. **The calculated band structures of Sr_2MnSbO_6 with different Hubbard parameter U**

Figure S18. The calculated band structures of Sr_2MnSbO_6 with different Hubbard parameter U (U = 4, 6 and 8 eV, red for up spins and blue for down spins).

1. Rietveld refinement of ambient temperature neutron powder diffraction data

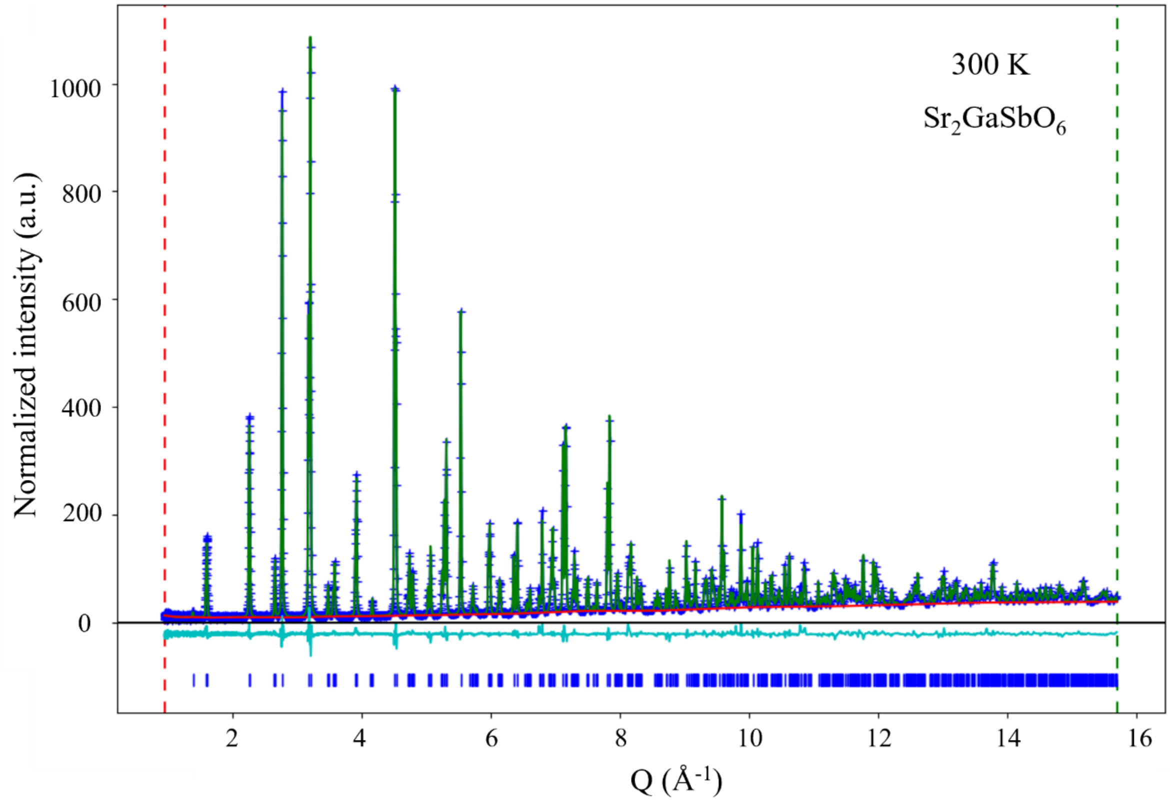

Figure S1. Observed (blue), calculated (green) and difference (cyan) plots from the Rietveld refinement of Sr_2GaSbO_6 (space group *I*4/*m*) against neutron powder diffraction data at 300 K.

Atoms	*x*/*a*	*y*/*b*	*z*/*c*	S.O.F.	U_{iso} equiv. (Å^2)
Sr1	0	0.5	0.25	1	0.00602
Ga1	0	0	0	1	0.00146
Sb1	0.5	0.5	0	1	0.00317
O1	0	0	0.2508(6)	1	0.00953
O2	0.2239(5)	0.2766(5)	0	1	0.00734

Sr_2GaSbO_6 space group *I*4/*m* (#87)
Formula weight: 462.72 g mol^{-1}, Z = 2
a = 5.54090(11) Å, *c* = 7.90490(9) Å, Volume = 242.693(12) Å^3
Radiation source: time of flight neutrons
Temperature: 300 K
wR = 5.100%; *GOF* = 3.83

Table S1. Structural parameters and crystallographic positions from the refinement of neutron powder diffraction data collected from Sr_2GaSbO_6 at 300 K.

Table S2 Selected bond lengths for the double perovskites from the Rietveld refinement of the Neutron diffraction data.

Sr_2GaSbO_6			$Sr_2Ga_{0.9}Mn_{0.1}SbO_6$		
**Sr(1)**	O(1)	2.770(1) (Å) × 4	**Sr(1)**	O(1)	2.770(1) (Å) × 4
	O(2)	2.932(1) (Å) × 4		O(2)	2.944(1) (Å) × 4
	O(2)	2.641(1) (Å) × 4		O(2)	2.641(1) (Å) × 4
**Ga(1)**	O(1)	1.982(1) (Å) × 2	**Ga/Mn(1)**	O(1)	1.975(1) (Å) × 2
	O(2)	1.972(1) (Å) × 4		O(2)	1.972(1) (Å) × 4
**Sb(1)**	O(1)	1.971(1) (Å) × 2	**Sb(1)**	O(1)	1.993(1) (Å) × 2
	O(2)	1.968(1) (Å) × 4		O(2)	1.970(1) (Å) × 4

2. Scanning Electron Microscope Characterization

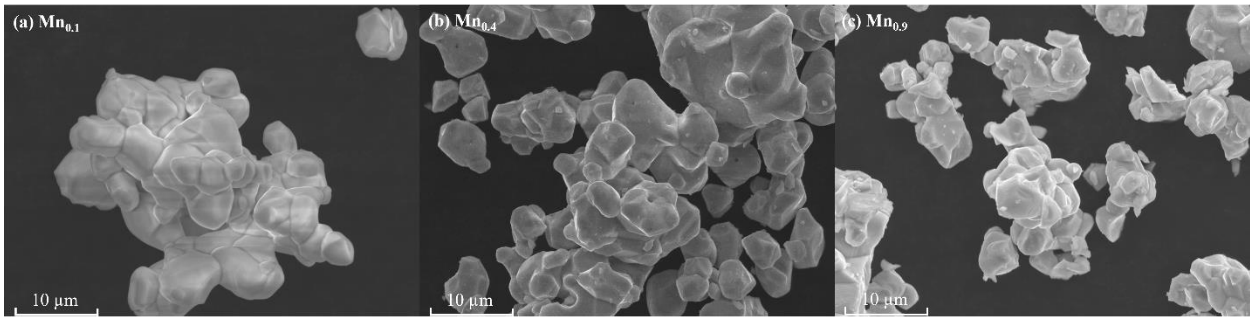

Figure S2. The FEI XL30 field-emission gun scanning electron microscope (SEM) photos of particle morphology of (a) $Sr_2Ga_{0.9}Mn_{0.1}SbO_6$, (b) $Sr_2Ga_{0.6}Mn_{0.4}SbO_6$ and (c) $Sr_2Ga_{0.1}Mn_{0.9}SbO_6$.

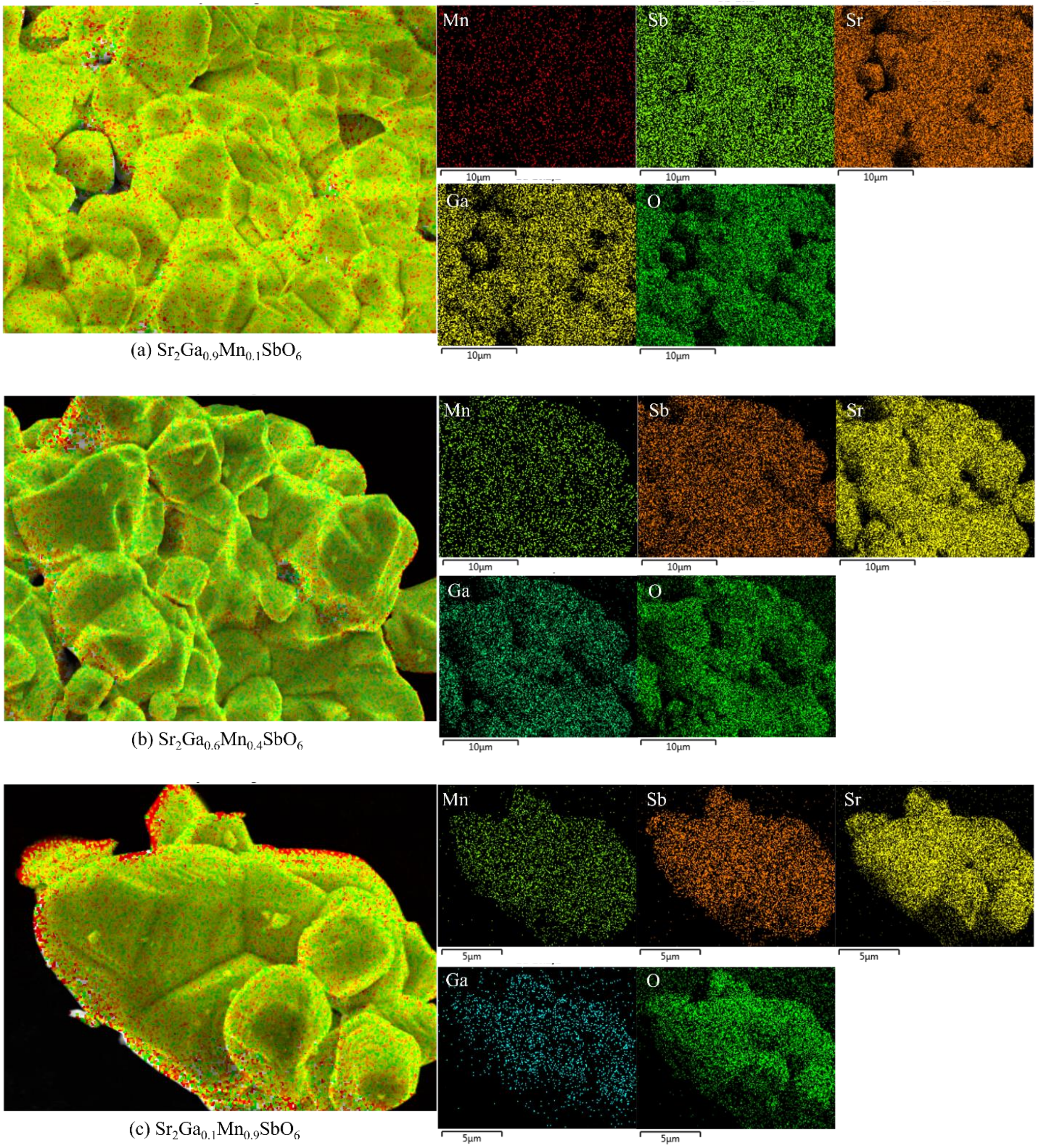

Figure S3. The scanning electron microscope (SEM) mapping results for (a) $Sr_2Ga_{0.9}Mn_{0.1}SbO_6$, (b) $Sr_2Ga_{0.6}Mn_{0.4}SbO_6$ and (c) $Sr_2Ga_{0.1}Mn_{0.9}SbO_6$.

3. Magnetization data for the $Sr_2Ga_{1-x}M_xSbO_6$ series (M = Cr/Mn/Fe) phases

Table S3. The Curie constant and Weiss temperature extracted from the fitting of paramagnetic susceptibility to the Curie-Weiss law, the observed effective moment per formula unit, and the calculated effective moment per formula unit predicted based on the spin-only formula for each composition of the Mn-doped double perovskite $Sr_2Ga_{1-x}Mn_xSbO_6$ series and single perovskite $LaGa_{1-x}Mn_xO_3$ series (scaled based on its equivalent double perovskite formula).

Composition	Curie constant C (cm^3 K mol^{-1})	Observed effective moment per formula unit $\mu_{eff.obs}$ (μ_B/f.u.)	Calculated effective moment per formula unit $\mu_{eff.cal}$ (μ_B/f.u.)	Weiss temperature θ (K)
**$Sr_2Ga_{1-x}Mn_xSbO_6$**				
x = 0.1	0.2993(2)	1.5473(5)	1.549	3.43(9)
x = 0.2	0.5978(5)	2.1868(9)	2.191	6.66(16)
x = 0.3	0.8875(8)	2.665(1)	2.683	8.04(17)
x = 0.4	1.303(2)	3.228(3)	3.098	9.23(22)
x = 0.5	1.580(2)	3.555(2)	3.464	16.80(26)
x = 0.6	1.987(3)	3.987(3)	3.795	25.15(31)
x = 0.7	2.379(4)	4.362(4)	4.099	38.27(40)
x = 0.8	2.843(5)	4.769(4)	4.382	42.70(44)
x = 0.9	3.317(8)	5.152(6)	4.648	57.10(60)
**$La_2Ga_{2-2x}Mn_{2x}O_6$**				
x = 0.1	0.6600(7)	2.298(1)	2.191	34.57(24)
x = 0.2	1.330(2)	3.262(3)	3.098	69.03(43)
x = 0.3	2.065(4)	4.064(4)	3.795	93.12(48)
x = 0.4	2.735(6)	4.678(5)	4.382	114.14(62)
x = 0.5	3.545(8)	5.327(6)	4.899	127.79(68)

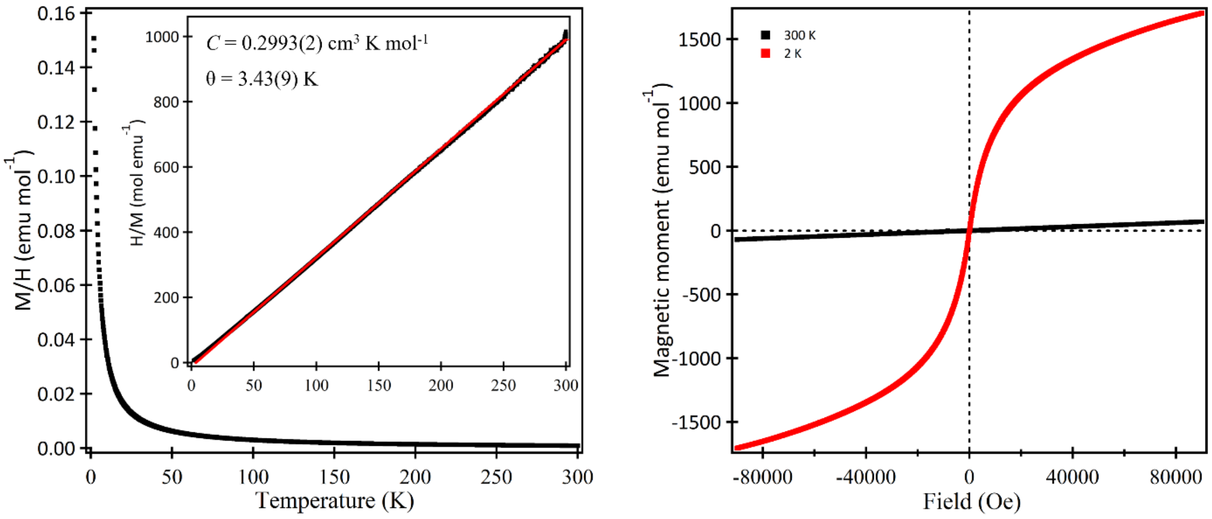

Figure S4. The magnetization data collected from $Sr_2Ga_{0.9}Mn_{0.1}SbO_6$.

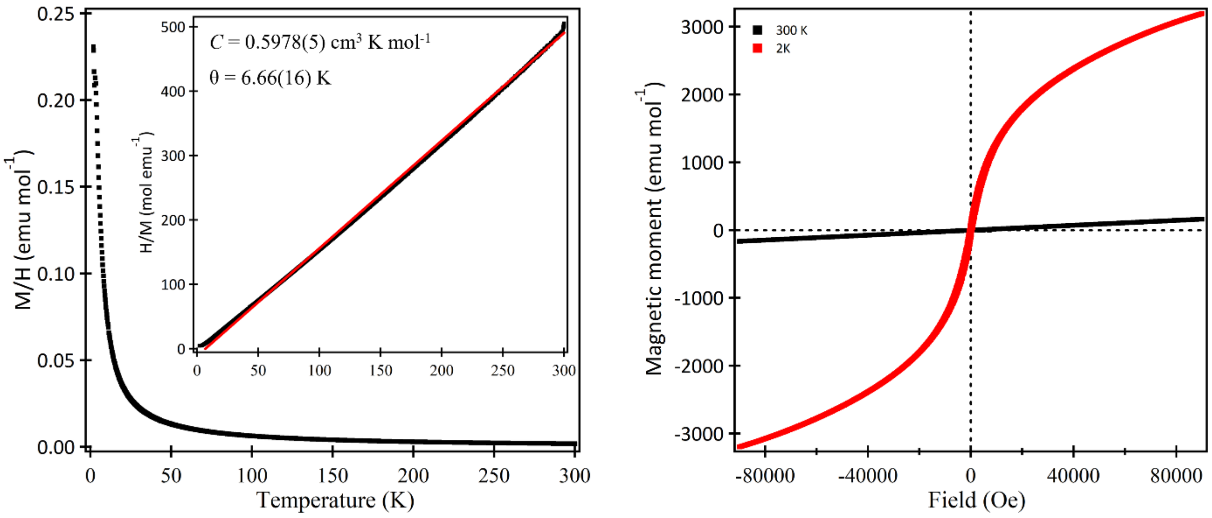

Figure S5. The magnetization data collected from $Sr_2Ga_{0.8}Mn_{0.2}SbO_6$.

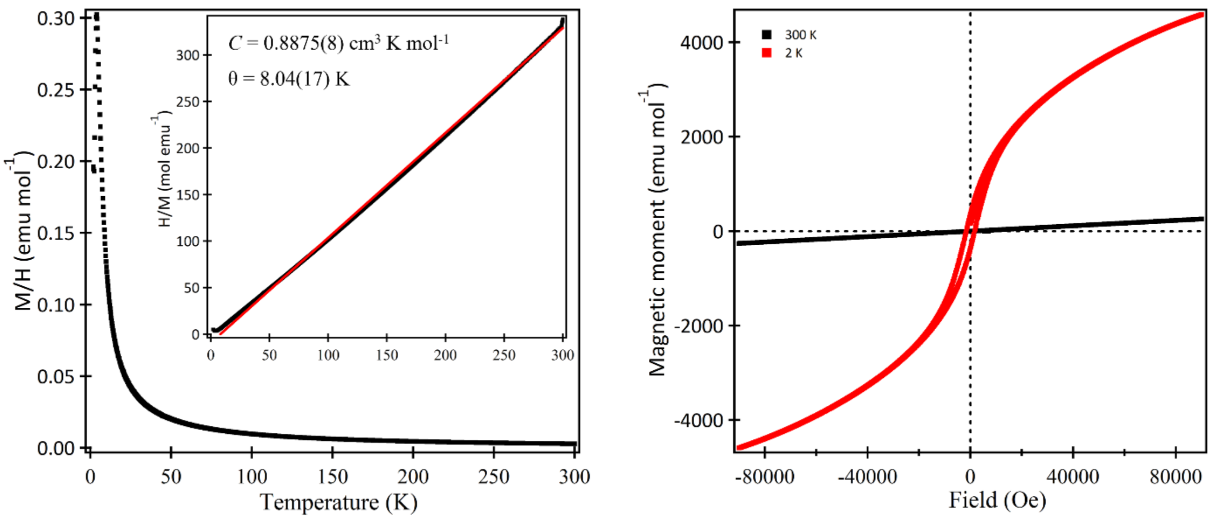

Figure S6. The magnetization data collected from $Sr_2Ga_{0.7}Mn_{0.3}SbO_6$.

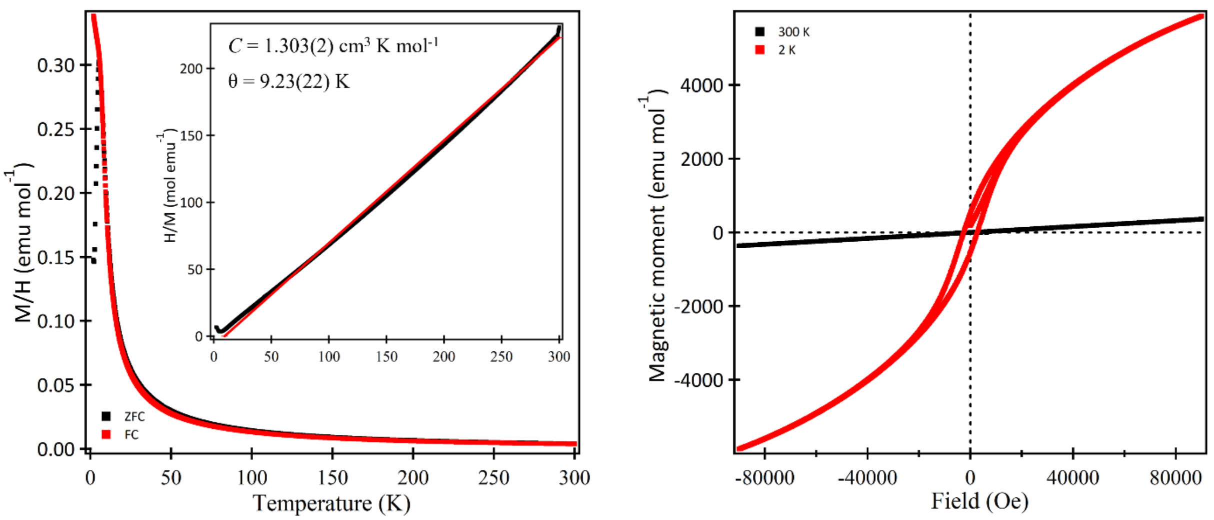

Figure S7. The magnetization data collected from $Sr_2Ga_{0.6}Mn_{0.4}SbO_6$.

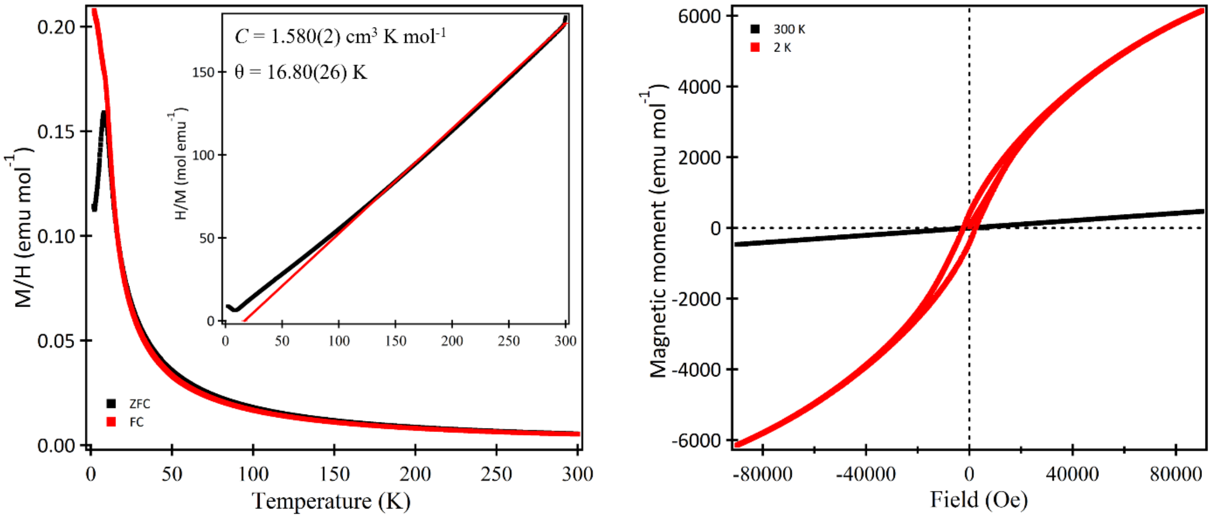

Figure S8. The magnetization data collected from $Sr_2Ga_{0.5}Mn_{0.5}SbO_6$.

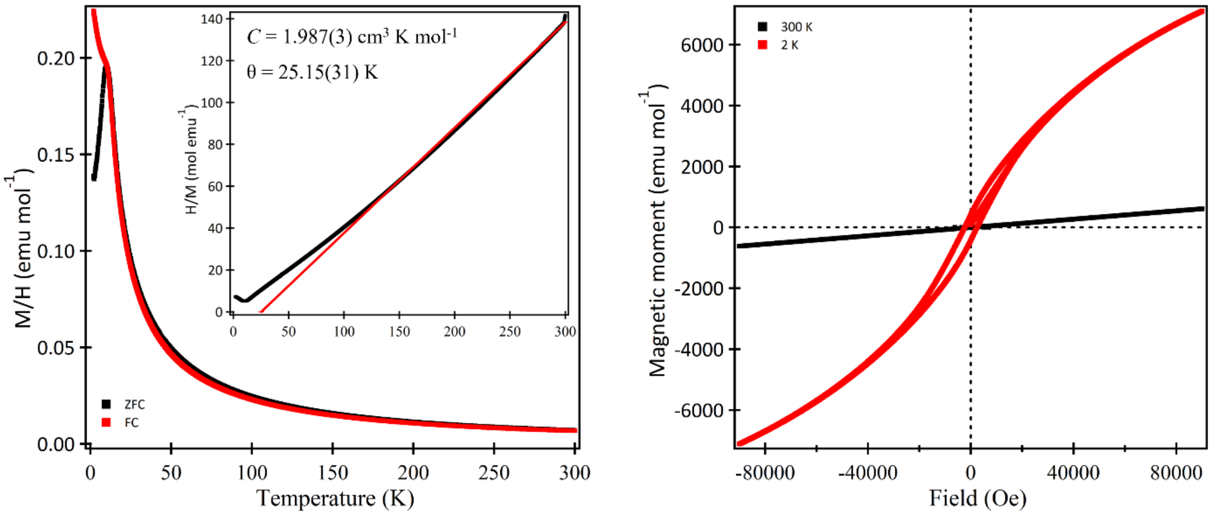

Figure S9. The magnetization data collected from $Sr_2Ga_{0.4}Mn_{0.6}SbO_6$.

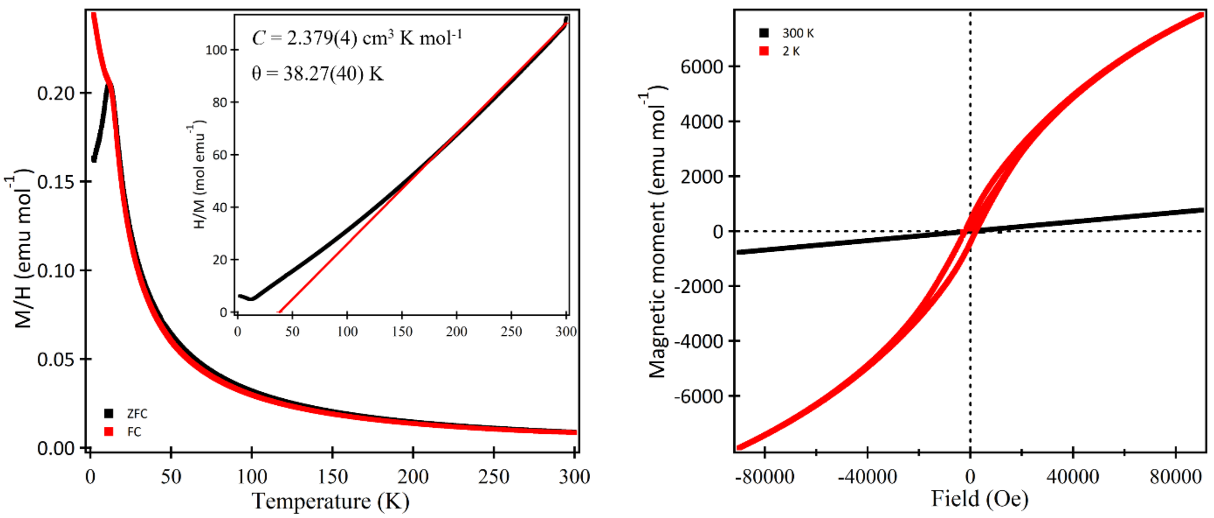

Figure S10. The magnetization data collected from $Sr_2Ga_{0.3}Mn_{0.7}SbO_6$.

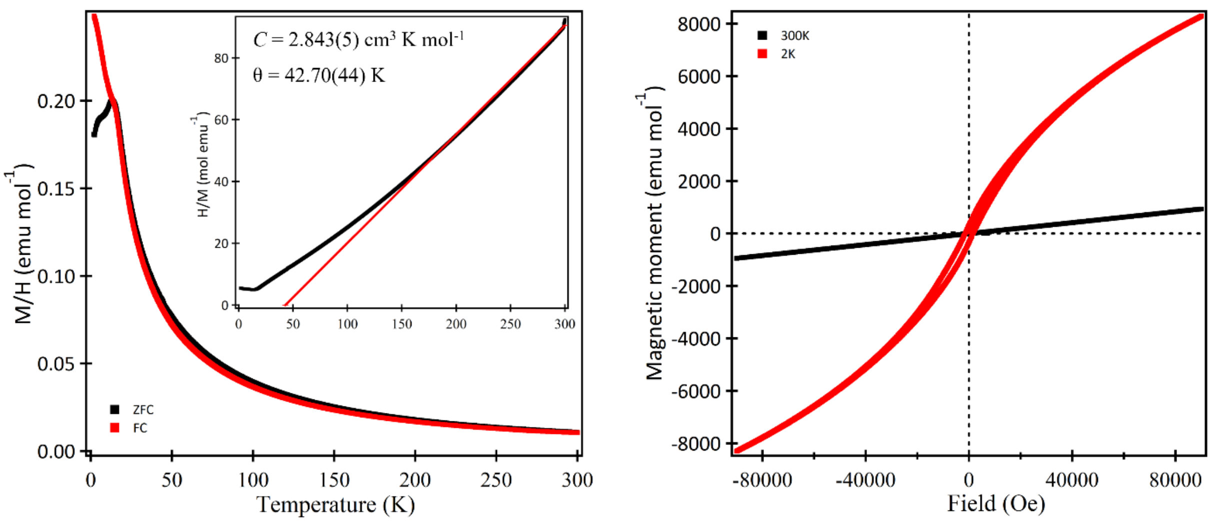

Figure S11. The magnetization data collected from $Sr_2Ga_{0.2}Mn_{0.8}SbO_6$.

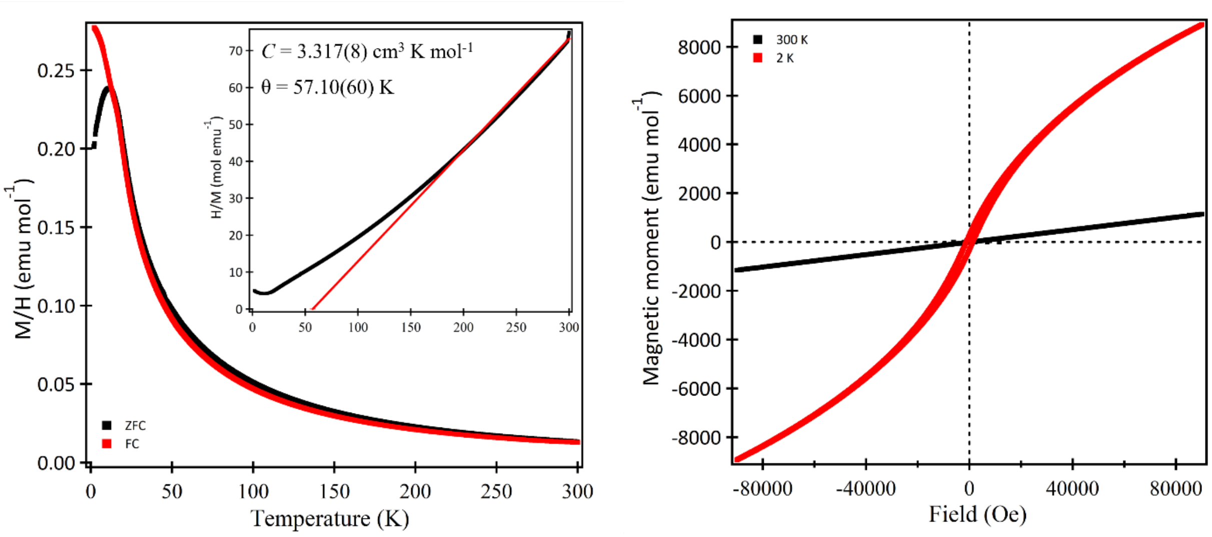

Figure S12. The magnetization data collected from $Sr_2Ga_{0.1}Mn_{0.9}SbO_6$.

4. Magnetization data collected from $La_2Ga_{2-2x}Mn_{2x}O_6$ series phases

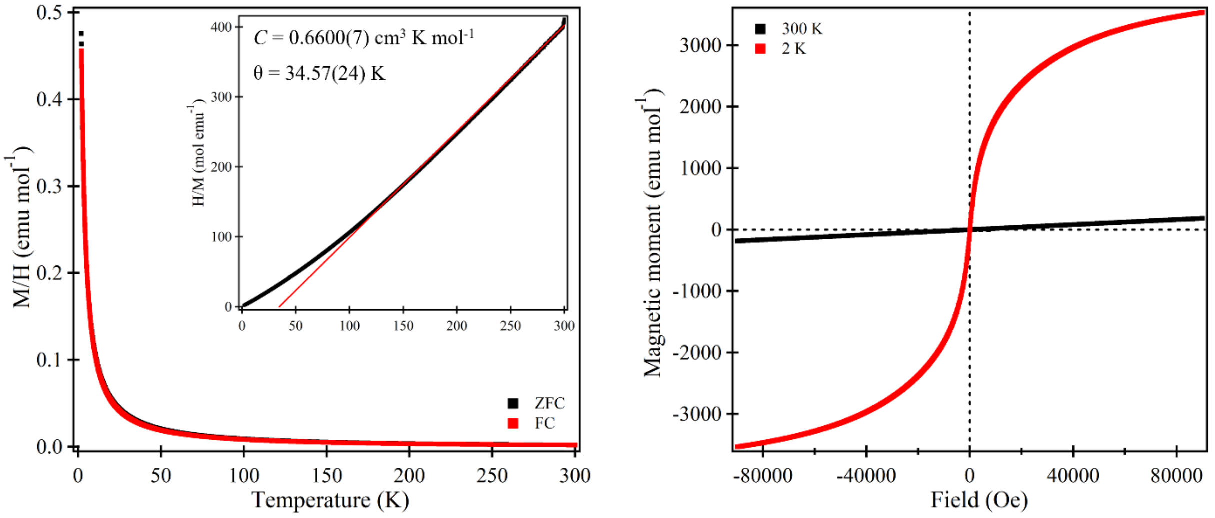

Figure S13. The magnetization data collected from $LaGa_{1.8}Mn_{0.2}O_6$.

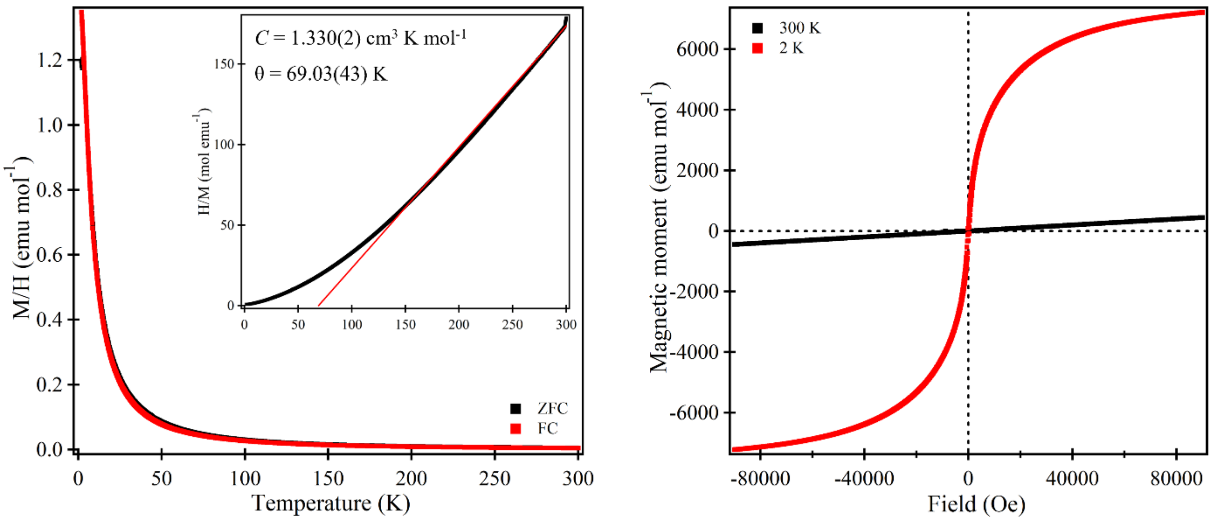

Figure S14. The magnetization data collected from $LaGa_{1.6}Mn_{0.4}O_6$.

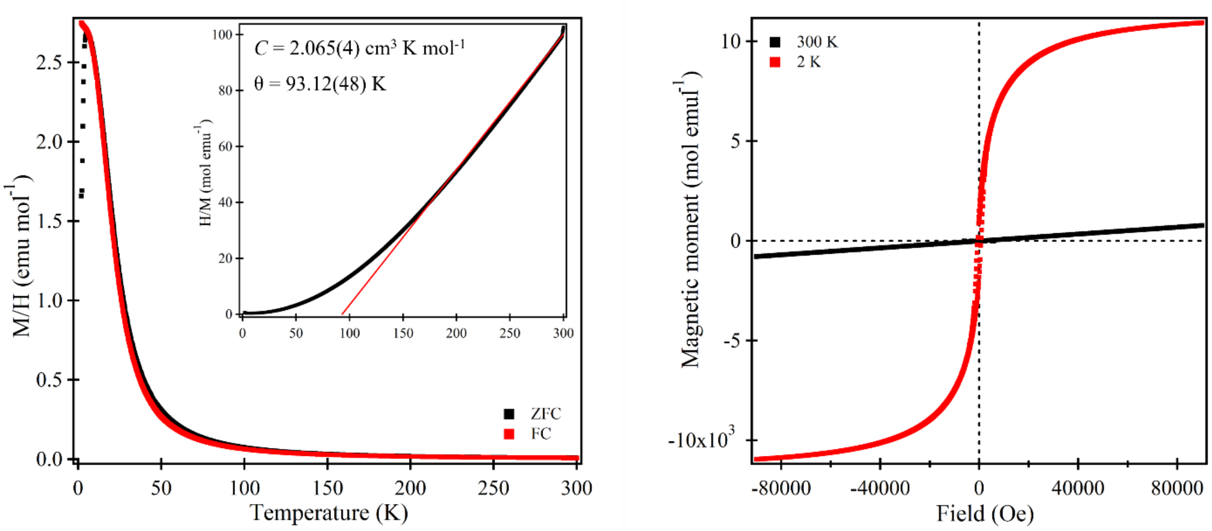

Figure S15. The magnetization data collected from $LaGa_{1.4}Mn_{0.6}O_6$.

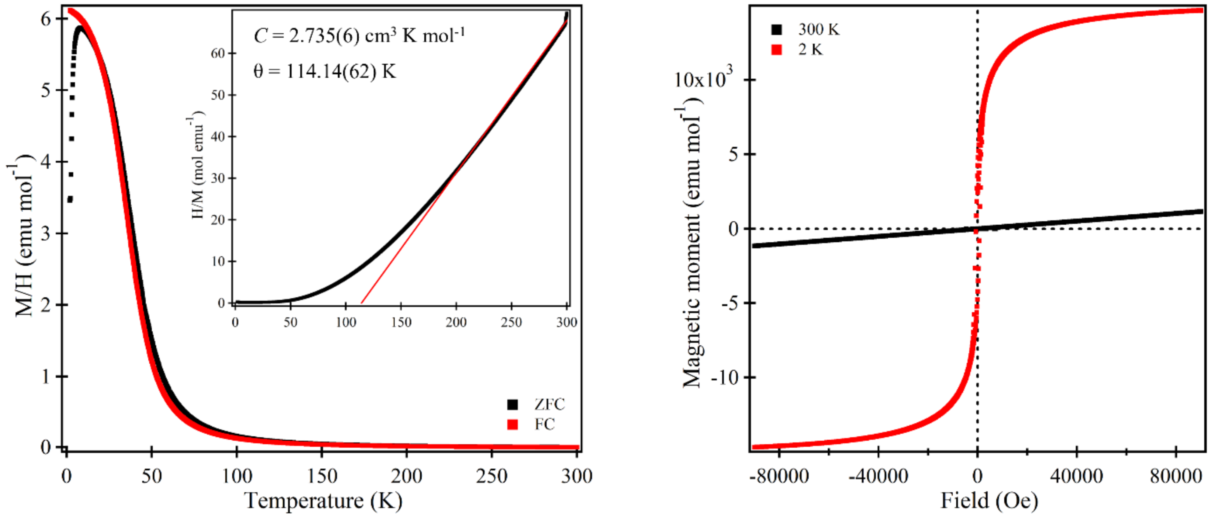

Figure S16. The magnetization data collected from $LaGa_{1.2}Mn_{0.8}O_6$.

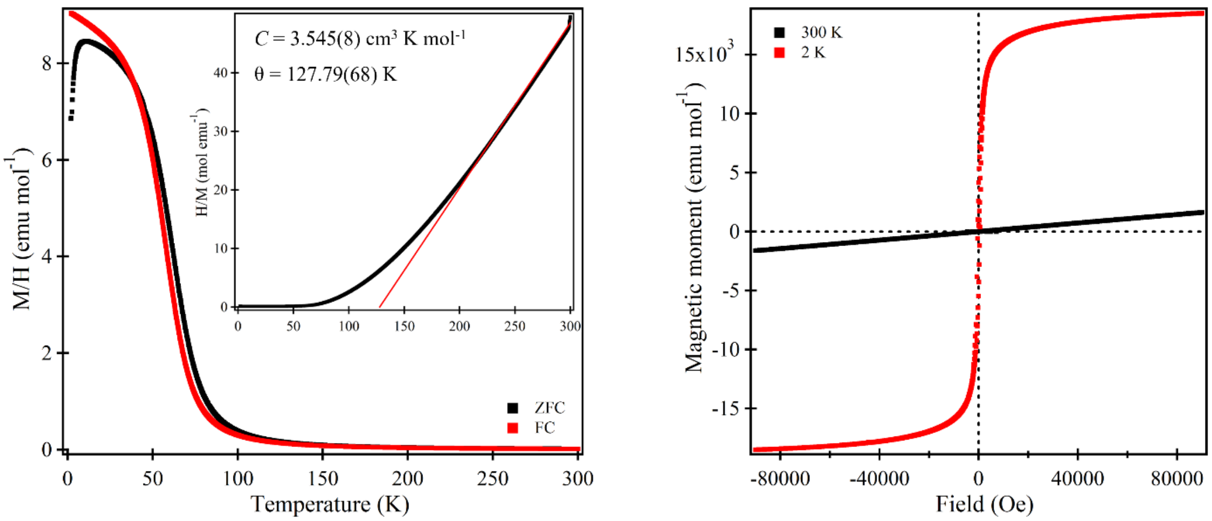

Figure S17. The magnetization data collected from $LaGa_{1.0}Mn_{1.0}O_6$.

5. The calculated band gaps for all the double perovskites prepared in this study

Table S4. The calculated band gaps from Tauc plot by using both indirect and direct transition equation for all the double perovskites prepared in this study.

Composition	Direct band gap (eV)		Indirect band gap (eV)	
**$Sr_2Ga_{1-x}Mn_xSbO_6$**				
$x = 0$	3.83		3.52	
	Gap 1	Gap 2	Gap 1	Gap 2
$x = 0.1$	2.99		2.27	1.15
$x = 0.2$	2.93		1.97	0.99
$x = 0.3$	2.22	1.50		0.90
$x = 0.4$	1.83	1.39		0.84
$x = 0.5$		1.16		0.61
$x = 0.6$		0.98		0.51
$x = 0.7$		0.92		0.49
$x = 0.8$	1.19	0.86		0.47
$x = 0.9$	1.22	0.75		0.46

Note: Gap 1 indicates the bandgap calculated from pseudo absorbance transition in the higher-energy regime, and Gap 2 indicates the bandgap calculated from pseudo absorbance transition in the lower-energy regime.

6. The calculated band structures of Sr_2MnSbO_6 with different Hubbard parameter U

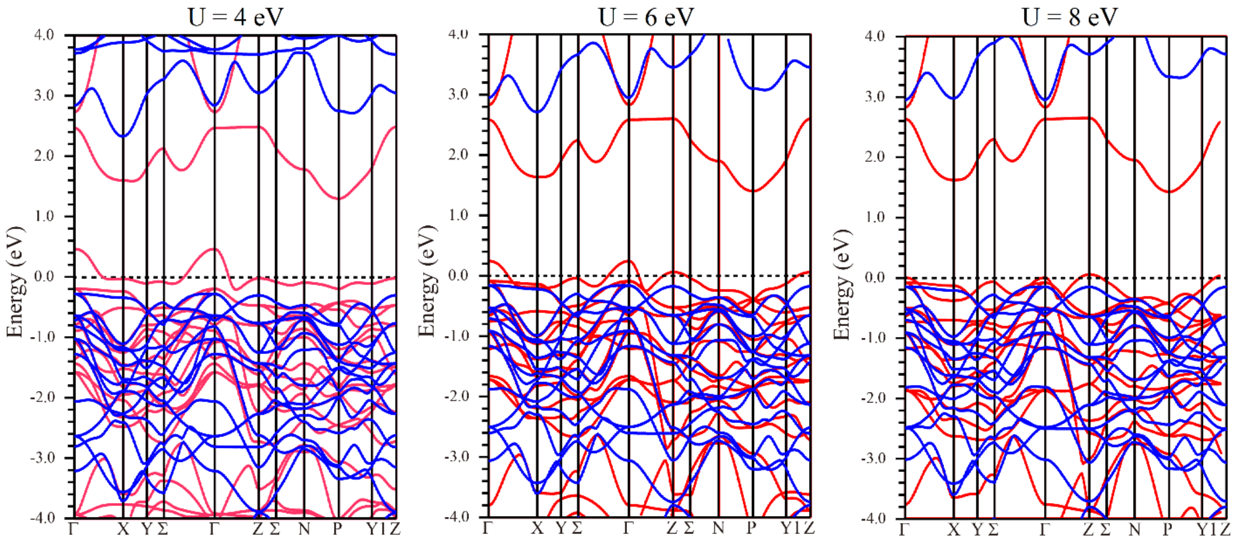

Figure S18. The calculated electronic band structures of Sr_2MnSbO_6 with different Hubbard parameters U (U = 4, 6 and 8 eV, red for up spins and blue for down spins).
